# Cognitive performance is linked to fitness in a wild primate

**DOI:** 10.1126/sciadv.adf9365

**Published:** 2023-07-12

**Authors:** Claudia Fichtel, Johanna Henke-von der Malsburg, Peter M. Kappeler

**Affiliations:** ^1^ Behavioral Ecology and Sociobiology Unit, German Primate Center, Leibniz Institute for Primate Research, Göttingen, Germany.; ^2^ Leibniz Science Campus “Primate Cognition”, Göttingen 37077, Germany.; ^3^ Department of Sociobiology/Anthropology, Johann-Friedrich-Blumenbach Institute for Zoology and Anthropology, University of Göttingen, Göttingen, Germany.

## Abstract

Cognitive performance varies widely across animal species, but the processes underlying cognitive evolution remain poorly known. For cognitive abilities to evolve, performance must be linked to individual fitness benefits, but these links have been rarely studied in primates even though they exceed most other mammals in these traits. We subjected 198 wild gray mouse lemurs to four cognitive and two personality tests and subsequently monitored their survival in a mark-recapture study. Our study revealed that survival was predicted by individual variation in cognitive performance as well as body mass and exploration. Because cognitive performance covaried negatively with exploration, individuals gathering more accurate information enjoyed better cognitive performance and lived longer, but so did heavier and more explorative individuals. These effects may reflect a speed-accuracy trade-off, with alternative strategies yielding similar overall fitness. The observed intraspecific variation in selective benefits of cognitive performance, if heritable, can provide the basis for the evolution of cognitive abilities in members of our lineage.

## Teaser

Wild gray mouse lemurs that perform better in a suite of cognitive tests enjoy greater survival.

## INTRODUCTION

Cognitive abilities vary considerably both within and among animal species (*1*–*3*). They guide behavioral decisions in many fitness-relevant contexts, such as homing, habitat and food selection, predator avoidance, mate choice, parental care, and the navigation of complex social challenges (*4*–*7*). Understanding the relationship between cognitive performance and fitness can help understand the factors surrounding cognitive evolution. Recent approaches toward explaining interspecific variation in cognitive abilities or the underlying relative brain size have traditionally taken a comparative approach to assess the potential role of ecological and social factors (*8*–*10*), but such studies can reveal neither how minds actually work (*11*) nor the evolutionary processes that shape intraspecific variation in cognitive abilities (*12*). Studies of interindividual variation in cognitive abilities of wild animals that show how they are associated with fitness are therefore required to determine to what extent cognitive abilities covary with each other as well as with the abilities to survive and to reproduce successfully (*3*, *13*).

The first step in investigating fitness consequences of cognitive variation is to establish whether particular cognitive abilities are critical for survival, reproductive success, or both (*3*). This approach is challenging because it requires both an estimate of cognitive performance and fitness proxies of the same individuals (*6*, *7*, *14*, *15*). The available studies suggest that both natural and sexual selection may act upon interindividual variation in some cognitive abilities, but these studies differed widely in the kinds and the number of cognitive tests as well as the fitness proxies used, and they revealed mixed results (*6*, *15*–*17*). Some studies found a positive link between cognitive performance and mate choice (*15*), reproductive success (*7*, *14*, *18*, *19*), or survival (*20*, *21*). Thus, differential fitness resulting from heritable variation in cognitive performance suggests that cognitive traits can evolve (*22*). However, in other studies, no or a negative link between cognitive performance and one of these fitness proxies has been reported (*18*, *23*–*26*). This outcome might be due to the fact either that there is a trade-off between the energetic costs of enhanced cognitive abilities and somatic maintenance or reproduction (*23*, *26*–*28*) or that the applied cognitive tests did not capture ecologically relevant cognitive traits (*21*).

In principle, selection may act upon different cognitive abilities separately or jointly as part of a general cognitive ability (*7*, *26*). The presence of a general intelligence factor (*g*) has been suggested for humans, but its existence remains debated in animals (*29*–*31*). One key finding supporting the existence of *g* are the uniformly positive correlations among different cognitive traits (*29*). To assess *g* in animals requires the establishment of a psychometric test battery encompassing several cognitive tasks tapping into different cognitive domains (*13*, *30*). If all tasks in the test battery address the same domain, however, they may reflect the same underlying cognitive mechanisms and the positive correlations indicative of a *g* may arise spuriously (*29*, *30*). Designing a suitable test battery that targets specific cognitive traits is challenging because tasks might be too much alike or associated with similar underlying motivations involved in solving a task, such as an inhibitory component in reversal learning or detour tasks (*2*, *30*, *32*). Moreover, identifying domains in animal cognition is not always straightforward, with some authors classifying spatial reversal learning tasks as spatial cognition, whereas others stress their inhibition component (*29*). Animals may recruit several specific cognitive abilities to solve a particular task, and different subjects or species may even enlist a different set of cognitive abilities (*29*, *33*, *34*).

The link between a general intelligence factor and proxies of fitness has been mainly studied in wild birds (*7*, *26*, *35*). In Western Australian magpies (*Gymnorhina tibicen dorsalis*), performance in associative and reversal learning, inhibitory control, and spatial memory correlated positively with each other, indicating the existence of a *g*, and females that had a higher *g* score enjoyed greater reproductive success (*7*). In contrast, in male spotted bowerbirds (*Gymnohina tibicen dorsalis*) and Arabian babblers (*Turdoides bicolor*), variation in a general intelligence factor did not predict reproductive success (*26*, *35*). In other studies, performance across cognitive tasks did not positively correlate with each other, indicating that the used cognitive tasks addressed abilities in different cognitive domains (*36*, *37*). Although cognitive functions are attributable to isolated operations of single brain areas, it is well established that cognition results from dynamic interactions of distributed brain areas operating in large-scale networks (*38*). Thus, even if there is domain-specific learning, it does not necessarily mean that cognitive problems are solved by solely activating these domain-specific brain areas. Hence, cognitive abilities work in concert, with several cognitive abilities potentially being involved in solving these “human-designed domain specific tasks.” Accordingly, a composite cognitive score that weighs performance across tasks equivalently might reflect this ability. Such a composite score has been used in studies of satin bowerbirds (*Ptilonorhynchus violaceus*) and male budgerigars (*Melopsittacus undulatus*), where it correlated positively with reproductive output (*36*, *37*). Therefore, independent of how cognitive performance has been assessed, either in single tests or via a *g* or composite score, there is suggestive evidence that cognitive performance tends to be associated with differential fitness in the reproductive domain.

Because of their relatively large brains, socially complex societies, and advanced cognitive abilities, primates have traditionally been a primary target to investigate the evolution of intelligence (*39*). However, although some primate populations have been studied for decades and their cognitive abilities are now being studied in the wild (*40*–*42*), the link between intraspecific variation in cognitive performance and fitness has so far only been assessed in one preliminary study of a wild primate: gray mouse lemurs (*Microcebus murinus*) (*17*). Performance in problem-solving and spatial memory was not associated with short-term survival, but problem-solving abilities predicted body condition. Because of this mixed support for a link between cognition and fitness, we expanded our preliminary study by estimating survival directly in a much larger sample and by establishing a battery of cognitive tests of multiple, ecologically relevant cognitive abilities (*34*, *43*). Specifically, we tested problem-solving abilities, spatial memory, inhibitory control, and causal understanding in separate tests. These domain-general tests are thought to capture cognitive abilities that play a pivotal role in various fitness-related behaviors (table S1). Moreover, because cognitive performance may also be influenced by intrinsic individual traits (*44*), we additionally conducted two personality tests—an open field test to assess exploration and a novel object test to assess neophilia—to control for potential noncognitive confounds.

Gray mouse lemurs are small nocturnal primates, endemic to Madagascar, that have become a model species in genetics, biomedical studies, and cognition (*45*, *46*). At our study site in Kirindy Forest, these ecological generalists face a full set of natural predators and competitors (*47*), have a median life span of 3 years (*48*), and are resilient to repeated testing in temporary captivity (*43*). In the present study, we first determined whether cognitive performance covaries across tasks. Next, we investigated whether cognitive performance is influenced by individual traits, such as age, sex, or personality. We then examined whether body mass, which is an established predictor of survival in gray mouse lemurs (*48*, *49*), is best predicted by cognitive performance, personality, age, sex, or rainfall in the year of testing (as a proxy for food availability). Last, we investigated the relative importance of cognitive performance and personality for predicting survival by controlling for other factors, namely, body mass, sex, age at testing, and food availability. We approximated lifetime food availability by calculating the mean monthly rainfall an individual experienced during its life. In contrast to most previous studies, we used lifetime survival as our main fitness proxy to contribute an additional perspective on the cognition-fitness link and because individuals with greater survival usually also have higher lifetime reproductive success (*21*).

## RESULTS

**Fig. 1.**
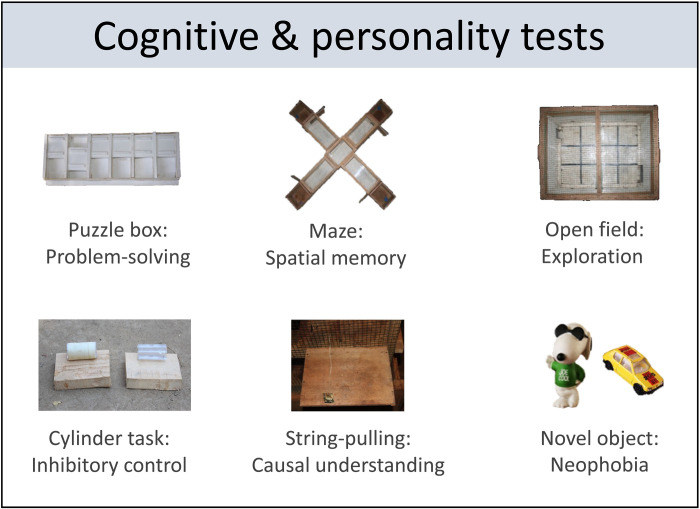
Test apparatuses for cognitive and personality tests. Test apparatuses that were used to investigate performance in the four cognitive and two personality tasks.

We conducted experiments using wild-caught mouse lemurs kept in temporary confinement for a maximum of three consecutive nights per test session. We quantified the cognitive performance of 198 individuals that reached a mean age of 1.94 years (range, 0.27 to 8.83 years) in tests designed to assess problem-solving abilities, spatial memory, inhibitory control, and causal understanding (Fig. 1; movies S1 to S4). We also conducted two standard personality tests: an open field and a novel object test (Fig. 1; movie S5). Because all six tests could not be performed in a single session, individuals had to be recaptured, resulting in a total of 194 individuals who performed both personality tests, of which 130 individuals performed four, 17 individuals three, 32 individuals two, and 15 individuals one cognitive test.

### Performance across tasks and composite cognition score

**Fig. 2.**
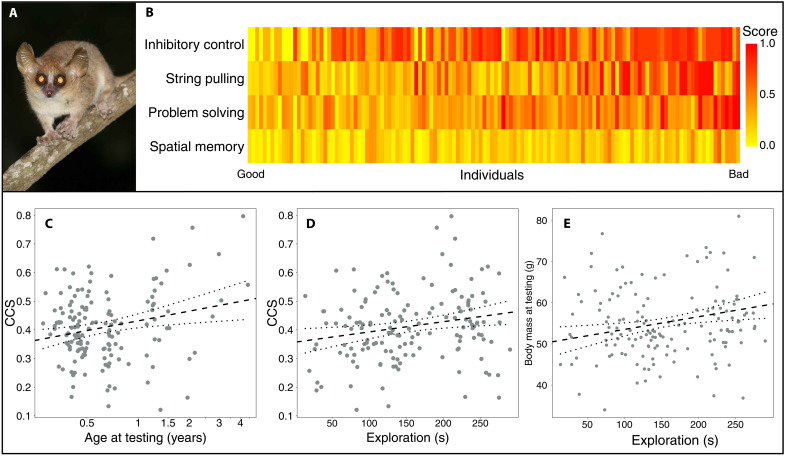
Variation in cognitive performance across tasks. (**A**) Gray mouse lemur. (**B**) Performance in single tests of each individual that participated in all four tests (*N* = 130). Yellow colors and lower numbers indicate better performance; red colors and higher numbers indicate poorer performance. (**C**) Composite cognition score (CCS) as a function of age at testing [linear model (LM): *N* = 147, *P* = 0.004] and (**D**) exploration (LM: *N* = 147, *P* = 0.010). (**E**) Body mass as a function of exploration (LM: *N* = 147, *P* = 0.006). Dashed lines indicate regression lines with corresponding 95% confidence interval (CI). Photo credit: C. Fichtel.

Individual mouse lemurs varied in longevity, body mass, as well as in performance in personality and cognitive tests (fig. S1). Repeatability in performance was moderate, except for problem-solving abilities (see Materials and Methods). Individual performance did not correlate positively across tasks (table S2), suggesting that no underlying general intelligence factor can be derived from these test scores. Therefore, we derived a composite cognition score (CCS) by first transposing the different measures of performance to a value between 0 and 1 (fig. S2), with lower values representing better performance (Fig. 2B). Next, we calculated a mean score for individuals that participated in at least three cognitive tests (*N* = 147; Fig. 2B). On average, mouse lemurs performed best in the spatial memory task (mean ± SD: 0.20 ± 0.15; Fig. 2B), equally well in the problem-solving (0.44 ± 23) and string-pulling tasks (0.44 ± 28), and were poorest at exhibiting inhibitory control (0.57 ± 0.28).

### Influence of individual characteristics on cognitive performance

We found that CCS covaried with age at testing and exploration (open field test), but not with neophilia (novel object test) and sex (Table 1A; *F* test: full-null model comparison: *F* = *F* = 3.70, *P* = 0.007). Specifically, older individuals and more explorative individuals performed on average worse than younger and less explorative individuals (Fig. 2, C and D).

**Table 1.** Summary statistics. Results of the linear models (LMs) estimating (A) the influence of age at testing, sex, exploration, and neophilia on cognitive performance [composite cognition score (CCS)] (*N* = 147); (B) the influence of sex, age at testing, CCS, exploration, neophilia, and rainfall on average body mass at testing (*N* = 147); and (C) the influence of sex, age at testing, performance in the problem-solving, spatial memory, causal understanding, and inhibitory control tasks, as well as exploration, neophilia, and rainfall on average body mass at testing (*N* = 147).

Response variable	Predictor	Estimate	SE	*P*
(A) CCS	Intercept	0.43	0.01	*
	Sex (male)^†^	−0.02	0.02	0.310
	Age at testing	0.03	0.01	0.004
	Exploration	0.03	0.01	0.010
	Neophilia	−0.01	0.01	0.514
(B) Body mass at testing and CCS	Intercept	56.38	0.82	*
	Sex (male)^†^	−1.88	1.07	0.082
	Age at testing	6.24	0.61	<0.001
	CCS	−0.05	0.75	0.948
	Exploration	2.28	0.83	0.007
	Neophilia	−0.67	0.57	0.242
	Rainfall	−1.29	0.80	0.111
(C) Body mass at testing and single cognitive tests	Intercept	54.16	2.54	*
	Sex (male)^†^	−0.66	1.21	0.583
	Problem-solving score	2.17	2.90	0.457
	Spatial memory score	2.05	4.58	0.655
	Causal understanding score	−2.57	2.51	0.309
	Inhibitory control score	1.92	2.11	0.365
	Exploration	2.44	0.95	0.012
	Age at testing	6.79	1.21	<0.001
	Neophilia	−1.02	0.64	0.117
	Rainfall	−1.00	0.96	0.299

*Not shown as has no meaningful interpretation.

†Females as reference category.

### Factors influencing body mass

On average, mouse lemurs had a body mass of 55.9 ± 9.4 g (mean ± SD). We found that average body mass at testing was best predicted by age and exploration, with older and more explorative individuals having a higher body mass (Table 1B and Fig. 2E; *F* test: full-null model comparison: *F* = 18.8, *P* < 0.001). However, CCS, neophilia, and rainfall in the year of testing were not associated with body mass. Similarly, average body mass at testing was not predicted by performance in single cognitive tests, but instead by exploration and age (Table 1C; *F* test: full-null model comparison: *F* = 10.65, *P* < 0.001).

### Factors influencing survival

**Fig. 3.**
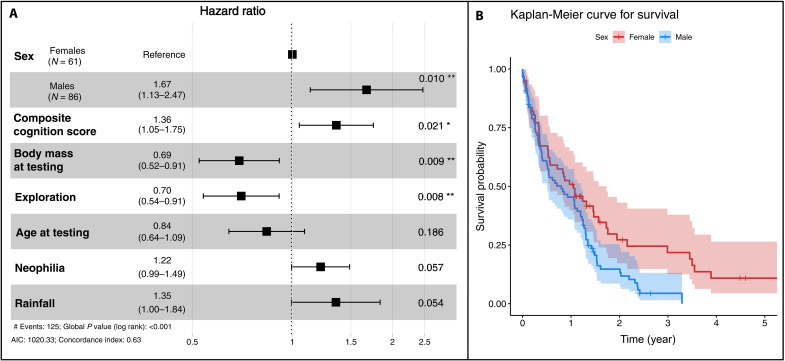
Survival analysis. (**A**) Forest plot depicting the hazard ratio and 95% confidence interval (CI) of years of survival after testing as a function of sex, composite cognition score (CCS), exploration, neophilia, body mass and age at testing, and average lifetime rainfall (Cox proportional hazard model, *N* = 147). (**B**) Kaplan-Meier curve for survival after testing.

We found that survival after testing was predicted by sex, CCS, body mass, and exploration (Fig. 3, A and B, and Table 2A; Wald test: χ^2^ = 29.39, df = 7, *P* < 0.001). Males and individuals with a higher CCS (i.e., indicating poorer cognitive performance) died sooner. In contrast, individuals with a higher body mass and those that were more explorative survived for longer. Age at testing, neophilia, and rainfall were not significant predictors of survival. Similarly, the Cox proportional hazard model including performance in single tests revealed that survival after testing was significantly predicted by sex and body mass, but only by trend by performance in the problem-solving and inhibitory control task as well as by exploration (Table 2B; Wald test: χ^2^ = 23.59, df = 10, *P* = 0.009). Males and, by trend, individuals with a less good performance in the problem-solving and inhibitory control task died sooner. Heavier and, by trend, more explorative individuals lived longer, however. Neither performance in the spatial memory or causal understanding task nor age at testing, neophilia, and rainfall did covary with survival after testing (Table 2B).

**Table 2.** Survival analysis. Results of the Cox proportional hazard model estimating (A) the influence of sex, composite cognition score (CCS), average body mass at testing, exploration, age at testing, neophilia, and average rainfall on survival after testing (*N* = 147) and (B) the influence of sex, performance in the problem-solving, spatial memory, causal understanding, and inhibitory control tasks, as well as average body mass at testing, exploration, age at testing, neophilia, and average rainfall on survival after testing (*N* = 147).

Response variable	Predictor	Coefficient	SE	*P*
(A) Survival after testing and CCS	Sex (male)*	0.51	0.20	0.010
	CCS	0.31	0.13	0.021
	Body mass at testing	−0.37	0.14	0.009
	Exploration	−0.35	0.13	0.008
	Age at testing	−0.18	0.13	0.186
	Neophilia	0.19	0.10	0.057
	Rainfall	0.30	0.15	0.054
(B) Survival after testing and single cognitive tests	Sex (male)*	0.53	0.22	0.019
	Problem-solving score	0.23	0.12	0.061
	Spatial memory score	−0.05	0.11	0.665
	Causal understanding score	0.07	0.12	0.553
	Inhibitory control score	0.18	0.10	0.085
	Body mass at testing	−0.37	0.15	0.014
	Exploration	−0.27	0.15	0.070
	Age at testing	−0.06	0.16	0.701
	Neophilia	0.13	0.12	0.255
	Rainfall	0.22	0.17	0.182

*Females as reference category.

## DISCUSSION

We investigated the cognition-fitness link in a wild primate species by quantifying cognitive performance across four domain-general cognitive tasks and two personality tests. Performances across tasks did not correlate positively with each other, suggesting that there is no general intelligence factor underlying variation in individual performance in this species. The derived CCS covaried with age and exploration. Body mass, an important physical predictor of survival, covaried neither with the CCS nor with performance in single cognitive tests. However, body mass covaried with age at testing and exploration. In the survival analysis, we controlled for age, body mass, and rainfall to gauge the relative importance of these factors and cognitive abilities to predict survival. Survival was predicted by body mass, age, exploration, and CCS. Our findings therefore support a key prediction of the role of selection in driving cognitive evolution: Superior cognitive abilities are associated with tangible fitness benefits in gray mouse lemurs.

### CCS and factors influencing cognitive performance

As in many other animal species studied previously (*29*, *30*), we did not find evidence for the existence of a general intelligence factor. Among primates, there is contradictory support for a *g* factor across and even within species (*32*, *43*, *50*–*52*). In captive chimpanzees (*Pan troglodytes*), for example, a *g* factor derived from performance in tests of the Primate Cognition Test Battery has been reported in one (*51*), but not in another population (*50*). However, as research on general intelligence in nonhuman animals is still in its infancy, and the question of how best to develop robust domain-specific tasks and statistical methods to derive a *g* factor is still heavily debated (*29*–*31*, *53*), more research is needed for a better understanding of the evolution of general intelligence. Even so, cognitive abilities nevertheless work in concert, with several cognitive abilities potentially being involved in solving a given cognitive task (*29*, *33*, *34*, *38*), which may be assessed by a CCS that weighs performance across all tasks equivalently (*36*, *37*).

In mouse lemurs, this CCS was influenced by age and exploration, but not by sex or body mass, with older and more explorative individual performing less good. Cognitive senescence has been documented in captive mouse lemurs, with individuals being older than 5 years exhibiting deficits in memory, flexible learning, and spatial abilities (*54*). However, unlike their captive conspecifics, wild mouse lemurs do not seem to exhibit functional senescence (*48*). Thus, potential age effects in cognitive performance should be treated with caution because only few individuals reach such an old age in the wild; we tested overall only four individuals older than 5 years. A decline with age in a general cognition factor has also been reported in female Arabian babblers (*26*). Because older females produced more fledglings, there might be a trade-off between cognitive performance and reproductive success in this species (*26*).

Personality has been suggested to be one predictor of individual variation in cognitive performance, with personality types being linked to cognitive styles via a speed-accuracy trade-off (*55*). Fast animals that are more explorative, aggressive, and/or bolder take risks while gathering more short-term gains, whereas slow animals take time to make accurate inferences and decisions that are often safer, but associated with relatively low short-term gains (*55*). Mouse lemurs that were more explorative had a higher CCS and performed poorer in the cognitive tests, supporting the existence of this speed-accuracy trade-off. Body mass as a proxy for hunger and, thus, motivation to engage with the experimental apparatuses (*56*), as well as neophilia did not covary with CCS and did therefore not influence performance in cognitive tests.

### Factors predicting body mass

Body mass is one of the best predictors of longevity in several mammalian species (*57*). Body mass also best predicted mouse lemurs’ short-term survival (*48*, *49*). However, body mass was not predicted by CCS, but instead by age and exploration, with older and more explorative individuals having a higher body mass. Because explorative tendencies can affect foraging strategies (*58*, *59*), more explorative mouse lemurs may potentially gather more resources and might be better in optimizing their foraging strategies than less explorative individuals. Neophilia did not covary with body mass, paralleling results of earlier studies on personality and life-history trade-offs in mouse lemurs (*60*).

### Factors predicting survival

We found that survival was best predicted by body mass, sex, exploration, and CCS. Heavier and more explorative mouse lemurs lived longer after testing, whereas males and individuals that had a higher CCS, i.e., poorer cognitive performance, died sooner. In line with earlier studies in this population of mouse lemurs, body mass and sex predicted survival in the present large sample (*48*, *49*, *61*). Moreover, more explorative individuals lived longer, in echoing findings of a meta-analysis across animals ranging from insects to mammals (*62*). Whereas heavier and more explorative individuals lived longer, individuals that gathered more accurate information and exhibited better cognitive performance also lived longer. These patterns may reflect a potential speed-accuracy trade-off with alternative strategies yielding similar overall fitness (*55*).

Rainfall experienced during mouse lemurs’ lifetime until testing, neophilia, or age at testing did not influence survival. Because age at testing did not predict survival after testing, the effect of CCS on survival should not be driven by older individuals with poorer cognitive performance. Such an effect might arise if animals become less motivated with age to enter traps. However, mouse lemurs are generally very “trap-happy” (many individuals have been trapped >100 times), and it will now be interesting to determine whether cognitive test performance, exploration, or neophilia influence the probability of recapturing individuals (*63*, *64*).

The survival analysis including performance in single cognitive tests revealed that survival was predicted by trend in two (problem-solving and inhibitory control) out of the four cognitive tests. Animals that performed poorer in the problem-solving and inhibitory control task tended to die sooner, mirroring the results of the analysis using the CCS. Because animals may use several cognitive abilities to solve a given problem (*29*, *33*, *34*, *38*), we think it is reasonable to derive a CCS that weights several cognitive measures equally, although they are not positively interrelated, to operationalize overall cognitive performance in this small test battery. This approach has already been applied in budgerigars and satin bowerbirds (*36*, *37*) and was slightly adapted in the present study (see Materials and Methods). In our earlier study, performance in two cognitive tests (problem-solving and spatial memory) did not covary with survival (*17*), which might be due to the fact that we only considered short-term survival in a much smaller sample than in the present study.

### Cognition and fitness

Few previous studies have studied the link between cognitive performance and survival (*21*). Survival/life span is likely to be a better predictor of total fitness than assessing reproductive success by counting offspring during one or a few seasons, but it is far more challenging to measure. Furthermore, in promiscuous species without paternal care, such as most mammals, cognitive performance is challenging to link to variation in reproductive success because of sex-specific differential investment in reproduction. In addition, many previous animal studies investigating the effects of cognitive performance on fitness used relatively small sample sizes or indirect measures of mate preferences, which may explain why they found effects for one sex but not the other, or in only 1 year but not another, or for only some fitness proxies and not others [(*6*, *65*, *66*), but see (*26*)]. Studies on humans, in contrast, agree with the key results of our study: Cognitive function and body mass index were both independent predictors of mortality risk in a Chinese population (*67*), and intelligence scores and fertility of Swedish males were positively correlated (*68*).

Our study demonstrates that individual differences in cognitive performance can result in differential fitness, but the heritability of cognitive traits remains to be demonstrated. Individual variation in cognitive abilities can only drive adaptive brain size evolution when they cause improvements in fitness that more than compensate for the higher energetic costs of larger brains (*69*) associated with superior cognitive abilities (*70*). However, our results do not allow us to draw conclusions about whether selection acts on individual cognitive abilities or on groups of them through some kind of threshold or cumulative effects. Determining individual variation in brain size (*66*), the neural and developmental underpinnings of cognitive evolution (*46*), and the exact nature and strength of selection on cognitive abilities remain important next steps. Mouse lemurs, because of their tractability in the laboratory and our ability to monitor them in the field to obtain ecologically relevant measures of fitness, have the potential to be a primate model species for this endeavor (*41*, *46*).

## MATERIALS AND METHODS

### Study site and subjects

We conducted this study in Kirindy Forest, a dry deciduous lowland forest in central western Madagascar located within a 12,500-ha forest concession operated by the Centre National de Formation, d’Etudes et de Recherche en Environnement et Foresterie (CNFEREF) Morondava (*71*). We captured gray mouse lemurs (*N* = 198) in a population that has been regularly monitored since 1995 by capturing them on a nearly monthly basis as part of an ongoing long-term study (*61*, *72*, *73*). To do so, we baited Sherman live traps with banana, set them at dusk at trail intersections, and collected them at dawn. Captured mouse lemurs were brought to the nearby field station and individually identified. All individuals were weighed. If they were newly captured, they were briefly restrained with 0.6 μl of ketamine (50 mg/ml) per 1 g body mass to mark them individually with a subdermal microtransponder (Trovan, Usling, Germany) and subjected to several standard morphometric measures, including body mass (*72*). We estimated an individual’s age by determining the number of days between birth and the date of the respective experimental test. We set an individual’s birth date to the modal birth date January 1st of the year of its first capture (*72*). To define death operationally for individuals not recaptured for longer periods, we determined the 95th percentile of the frequency distribution of 10,936 intercapture intervals recorded between 1995 and 2017 as a cutoff point. Accordingly, study subjects were operationally considered dead if they were not recaptured within 161 days. Because mouse lemurs exhibit “trap-happiness” and enter traps regularly (*61*), we do not assume that some individuals were mistakenly recorded as dead because of individuals becoming trap shy.

### Housing and experimental procedure

Experiments were conducted between 2015 and 2019 by keeping animals in temporary short-term captivity at the field station during the dry season (March to November). Mouse lemurs were housed in cages of 80 cm × 80 cm × 80 cm equipped with a nest box, several branches, an experimental platform, and ad libitum access to water. After testing, mouse lemurs were fed with insects and banana. We kept animals for a maximum of three nights, after which they were released back at their site of capture. In total, we tested up to 198 mouse lemurs per task in a total of 1038 tests (*17*, *43*). Because the complete experimental test battery was usually not completed during one test session comprising three consecutive nights, we selectively recaptured subjects after they had spent at least three nights back in the forest. In general, mouse lemurs respond to capturing with a short-term increase in fecal glucocorticoid metabolite concentrations (fGCMs) on day 1 or 2 after capture and a decrease to baseline levels on day 2 or 3 after capture (*48*). However, there was no evidence for long-term consequences of repeated captures on the animal’s stress physiology (*48*). Although we cannot rule out that the initial increase in fGCM levels influenced cognitive performance, it should, however, be similar across individuals because they were all treated in the same way. Because mouse lemurs were only tested when they voluntarily entered and explored the experimental platform, and the majority of individuals participated in the cognitive tests already in the first night after capture, we are confident that performance in cognitive tests was at most only marginally influenced by potential physiological stress and that this putative effect did not introduce any systematic bias.

Testing started between 06:00 and 07:00 p.m. under red light conditions, whose wavelength is not visible for the dichromatic mouse lemurs (*74*), and ended when the motivation of the animals ceased. The experimental test battery comprised two personality tests (an open field and a novel object test) and four cognitive tests (a problem-solving, spatial memory, inhibitory control, and causal understanding task), for which we used small pieces of banana as food rewards (Fig. 1). The order of the tests was randomized and counterbalanced among subjects. Before any experimental session, we cleaned the experimental platform, the test apparatus, or the respective arena for the open field test and plus maze with 70% ethanol to remove any odor cues. At the beginning of each test, we lured mouse lemurs with a stick covered with banana to the starting position at the opposite end of the experimental platform. Each experimental test session was videotaped (Sony HDR-CX 240) and later analyzed using BORIS (*75*). For each test, 10% of the videos were double-coded by a second observer naïve to the research question, resulting in a mean interobserver reliability of 95.9% [minimum: 80.2%, maximum: 100%; (*43*)]. Because it was logistically impossible to re-test all individuals, repeatability estimates were based on small subsamples. Repeatability was calculated by intraclass correlation coefficients.

### Ethical statement

The Malagasy Ministry of the Environment, the Mention Zoologie et Biodiversité Animale de l’Université d’Antananarivo and the CNFEREF Morondava (N°245/18/MEEF/SG/DGF/DSAP/SCP.re and N°47/18/MEEF/SG/DGF/DSAP/SCP.re), and the relevant German Animal Use and Care committees, i.e., the Animal Welfare Body of the German Primate Center, approved and authorized this research (E9-18).

### Personality tests

#### Exploration and neophobia

We assessed an individual’s explorative tendencies in an unknown environment, using an open field test with either a rectangular (80 cm × 60 cm × 60 cm; Fig. 1) or cylindric wooden arena (Ø 80 cm × 80 cm). After subjects entered the arena voluntarily, they were observed for 5 min exploring the arena. We used the duration subjects spent locomoting as measure for exploration (movie S5). To assess an individual’s neophilic tendencies, we introduced a novel object (either a plastic snoopy or a toy car; Fig. 1) directly after each open field test into the arena and measured how often they contacted the novel object within a 5-min test duration as a measure of neophilia (movie S5). Exploration (time spent locomoting: *N* = 83, ICC = 0.263) was moderately repeatable, and neophilia (number of contacts: *N* = 83, ICC = 0.028) was weakly repeatable.

### Cognitive tests

#### Problem-solving

To assess an individual’s problem-solving abilities, we presented a problem-solving box (6 cm × 12 cm; Fig. 1 and movie S1) consisting of six uniform wells (5 cm × 4.5 cm) that were each baited with a small piece of banana, which could be extracted by sliding a lid open. After the box was introduced onto the experimental platform within the subject’s cage, the animals had 20 min to extract the six rewards. As problem-solving abilities, we measured the mean time individuals needed to open the lids. In case an individual did not succeed at all, we set its success time to 20 min as the maximum test duration. Problem-solving abilities (solving efficiency: *N* = 20, ICC = 0.035) were weakly repeatable.

### Spatial memory

To assess an individual’s spatial memory, we set up a plus maze with four arms (40 cm × 17 cm × 17 cm) leading to four terminal boxes (20 cm × 17 cm × 17 cm; Fig. 1 and movie S2). Each end box contained a plastic lid in its back at the opposite side of its door, in which we placed a small piece of banana in case of the rewarding location, only visible from the door. For an initial familiarization trial, we baited three end boxes and released the subject at the fourth box, the start arm. The familiarization trial started when we opened the door of the start box and ended either when the subject had eaten all the three food rewards or after a maximum of 15 min. In that case, we repeated the familiarization trial until successful completion. For the actual test session (15 successive trials), only one end box (=goal box) was baited. As goal box, we chose the box in which we caught the animal at the end of the familiarization trial. Because only the goal box was baited during the test session, we placed a piece of banana peel that was out of reach for the subject on top of each end box to control for olfactory stimuli. Similar to the familiarization trial, a test trial started when we opened the door of the start box and ended as soon as the subject had retrieved the food reward in the goal box. We stopped a session when the subject did not exit the start box within 10 min and continued the session either later in the night or in the following night. We additionally cleaned the maze after every third trial. We rotated the goal boxes throughout the session to prevent the subject from following potential odor cues left inside the goal box. We counted how often a subject entered the wrong arm per trial and calculated the mean sum of errors over 15 trials. Spatial memory (mean number of errors: *N* = 19, ICC = 0.210) was moderately repeatable.

### Inhibitory control

To assess an individual’s inhibitory control, we conducted a detour-reaching task using the cylinder test design (Fig. 1 and movie S3). Before testing, we conducted a training session with an opaque cylinder. The food reward was placed in the center of the cylinder, invisible to the subject during its approach. To reach the reward, the subject had to take a detour and enter the cylinder by one of the open sides that were set in an 90° angle from the approach direction onto the experimental platform within the subject’s cage. We additionally removed odor cues after every fifth trial. For the actual testing session of 10 trials, we changed the cylinder to a transparent cylinder and repeated the cleaning every third trial, and the rest of the experimental setup was the same as in the training sessions. Using a transparent cylinder, the subject could see the charcoal-colored piece of banana while approaching the task. It could also smell the banana from the front through little holes in the center of the cylinder. Nevertheless, it had to inhibit an initial response to reach directly through the transparent barrier. Instead, it had to take a detour through one of the open sides to enter the cylinder to access the reward. We used the number of incorrect trials during a session of 10 trials as a measure for inhibitory control. Inhibitory control (number of errors: *N* = 8, ICC = 0.353) was moderately repeatable.

### Causal understanding

To assess an individual’s means-end understanding, we conducted a string-pulling task (Fig. 1 and movie S4). To this end, a cable tie was placed onto an external platform attached to the subject’s cage in front of the experimental platform within the cage. At the far end of the cable tie, we attached a piece of banana onto a small plate. The inner end of the cable tie reached 5 cm into the cage. Just before we placed the cable tie at its position, we lured the subject to the top wire in the center of the experimental platform. After positioning the cable tie, the subject had 20 min time to pull the end of the cable tie into reach to access the reward. We measured the success latency as time span between the response latency, i.e., first orientation toward the string, and accessing the reward. For subjects that did not succeed, we set the success latency to the maximum time of the trial (20 min) plus the response latency. Causal understanding (success latency: *N* = 14, ICC = 0.587) was repeatable.

### Statistical analyses

We conducted all statistical analyses in R (version 4.2.0, R Core Team, 2022). To assess repeatability in cognitive performance, we calculated intraclass correlation coefficients (i) by using the package “ICC” (*76*). We used Spearman rank correlations (ii) to investigate whether performances across cognitive tests correlated positively with each other. We derived a CCS akin to earlier studies on birds (*36*, *37*). They derived a CCS either by assigning performance with a rank of 1 being worst for a task and then calculating the mean (*37*) or by splitting the values for each measure of cognitive performance into four ranks (1 to 4), using the distribution quartiles as dividing points and adding them up (*36*). Because assigning ranks or splitting measures of cognitive performance in four ranks does not reflect the measured variance in cognitive performance, we applied an alternative method.

Specifically, we derived a CCS instead by first transposing the different measures of performance, i.e., durations or frequencies, from all individuals to a range between 0 and 1 {*x* transformed = [*x* − min(*x*)]/[max(*x*) − min(min)]}, which maintained the measured variation in performance (fig. S2). Then, we calculated a mean score for individuals that performed at least three cognitive tests (*N* = 147). Performances in the problem-solving, causal understanding, and inhibitory control task were log-transformed to achieve a less skewed distribution before transformation. We chose not to use a principal components analysis because performance in one or several cognitive tests may more heavily load on the derived principal components than others, weighting performance across tasks not equally (*31*, *36*).

To assess (iii) whether cognitive performance covaried with individual characteristics, we fitted a Gaussian linear model (LM) with the CCS score as response, sex, age at testing, exploration, and neophilia as predictors. To investigate (iv) whether body mass at testing was predicted by cognitive performance or personality, we estimated another LM by fitting average body mass as response. Because not all tests were performed in one test session and we had to recapture the animals later, we used the average body mass across test sessions in this model. CCS, rainfall in the year of testing as a proxy for food availability, and age at testing were included as predictors. We additionally fitted the same model by including each cognitive test separately instead of CCS. Before fitting LMs, we *z*-transformed quantitative covariates to a mean of 0 and an SD of 1 to facilitate interpretation of predictor estimates. We checked the model assumptions “absence of collinearity” using variance inflation factors [package “car”; (*77*)] and “absence of influential observations” using dfbetas and visually checked normally distributed and homogeneous residuals. To test the significance of the predictors as a whole, we compared all full models with the respective null model comprising only the intercept (*78*).

We estimated a Cox proportional hazard model [package “survival”; (*79*)] to estimate (v) whether survival after testing was predicted by body mass at testing, sex, CCS, exploration, neophilia, and mean lifetime rainfall as proxy for food availability. We chose years after testing as survival metric to account statistically for age effects on cognitive performance and survival because our sample consisted mainly of young individuals. We right-censored individuals that were still alive at the time of census (*N* = 22) or potentially dispersed (*N* = 10), i.e., males that were not recaptured up to an age of 8 months, the age at which they usually disperse (*80*). Before fitting, we *z*-transformed covariates to a mean of 0 and an SD of 1 to facilitate interpretation of predictor estimates. We checked for “absence of influential observations” using the package “survminer” and checked the violation of proportional hazards using the function “testph.” Again, we fitted the same survival model by including each cognitive test separately instead of the CCS performance. Because only 130 individuals performed all four cognitive tests, we additionally fitted a Cox proportional hazard model including CCS for these individuals separately (table S3).

## References

[1] N. J. Emery, N. S. Clayton, Comparative social cognition. Annu. Rev. Psychol. 60, 87–113 (2009). 18831684 10.1146/annurev.psych.60.110707.163526

[2] C. Rowe, S. D. Healy, Measuring cognition will be difficult but worth it: A response to comments on Rowe and Healy. Behav. Ecol. 25, –1298 (2014).

[3] J. Morand-Ferron, E. F. Cole, J. L. Quinn, Studying the evolutionary ecology of cognition in the wild: A review of practical and conceptual challenges. Biol. Rev. 91, 367–389 (2016). 25631282 10.1111/brv.12174

[4] D. Sol, T. Székely, A. Liker, L. Lefebvre, Big-brained birds survive better in nature. Proc. R. Soc. Lond. B. Biol. Sci. 274, 763–769 (2007). 10.1098/rspb.2006.3765 PMC2093983 17251112

[5] E. F. Cole, J. Morand-Ferron, A. E. Hinks, J. L. Quinn, Cognitive ability influences reproductive life history variation in the wild. Curr. Biol. 22, 1808–1812 (2012). 22940473 10.1016/j.cub.2012.07.051

[6] M. Cauchoix, A. S. Chaine, How can we study the evolution of animal minds? Front. Psychol. 7, 358 (2016). 27014163 10.3389/fpsyg.2016.00358 PMC4791388

[7] B. J. Ashton, A. R. Ridley, E. K. Edwards, A. Thornton, Cognitive performance is linked to group size and affects fitness in Australian magpies. Nature 554, 364–367 (2018). 29414945 10.1038/nature25503 PMC5815499

[8] R. I. M. Dunbar, S. Shultz, Understanding primate brain evolution. Philos. Trans. R. Soc. B. Biol. Sci. 362, 649–658 (2007). 10.1098/rstb.2006.2001 PMC2346523 17301028

[9] A. R. DeCasien, S. A. Williams, J. P. Higham, Primate brain size is predicted by diet but not sociality. Nat. Ecol. Evol. 1, 112 (2017). 28812699 10.1038/s41559-017-0112

[10] J. B. Smaers, R. S. Rothman, D. R. Hudson, A. M. Balanoff, B. Beatty, D. K. N. Dechmann, D. de Vries, J. C. Dunn, J. G. Fleagle, C. C. Gilbert, A. Goswami, A. N. Iwaniuk, W. L. Jungers, M. Kerney, D. T. Ksepka, P. R. Manger, C. S. Mongle, F. J. Rohlf, N. A. Smith, C. Soligo, V. Weisbecker, K. Safi, The evolution of mammalian brain size. Sci. Adv. 7, eabe2101 (2021). 33910907 10.1126/sciadv.abe2101 PMC8081360

[11] J. J. Bolhuis, Evolution cannot explain how minds work. Behav. Proc. 117, 82–91 (2015). 10.1016/j.beproc.2015.06.008 26091756

[12] A. Thornton, D. Lukas, Individual variation in cognitive performance: Developmental and evolutionary perspectives. Philos. Trans. R. Soc. Lond. B Biol. Sci. 367, 2773–2783 (2012). 22927576 10.1098/rstb.2012.0214 PMC3427550

[13] A. Thornton, How and why are some species so smart? A comment on Rowe and Healy. Behav. Ecol. 25, 1294–1295 (2014).

[14] R. C. Shaw, R. D. MacKinlay, N. S. Clayton, K. C. Burns, Memory performance influences male reproductive success in a wild bird. Curr. Biol. 29, 1498–1502.e3 (2019). 31006565 10.1016/j.cub.2019.03.027

[15] J. Chen, Y. Zou, Y.-H. Sun, C. ten Cate, Problem-solving males become more attractive to female budgerigars. Science 363, 166–167 (2019). 30630929 10.1126/science.aau8181

[16] V. Chantal, J. Gibelli, F. Dubois, Male foraging efficiency, but not male problem-solving performance, influences female mating preferences in zebra finches. PeerJ 4, e2409-13 (2016). 27635358 10.7717/peerj.2409 PMC5012330

[17] F. Huebner, C. Fichtel, P. M. Kappeler, Linking cognition with fitness in a wild primate: Fitness correlates of problem-solving performance and spatial learning ability. Philos. Trans. R. Soc. Lond. B Biol. Sci. 373, 20170295 (2018). 30104438 10.1098/rstb.2017.0295 PMC6107563

[18] E. F. Cole, J. L. Quinn, Personality and problem-solving performance explain competitive ability in the wild. Proc. R. Soc. Lond. B Biol. Sci. 279, 1168–1175 (2012). 10.1098/rspb.2011.1539 PMC3267141 21937498

[19] L. Cauchard, B. Angers, N. J. Boogert, M. Lenarth, P. Bize, B. Doligez, An experimental test of a causal link between problem-solving performance and reproductive success in wild great tits. Front. Ecol. Evol. 5, 107 (2017).

[20] C. Rochais, C. Schradin, N. Pillay, Cognitive performance is linked to survival in free-living African striped mice. Proc. R. Soc. Lond. B Biol. Sci. 290, 20230205 (2023). 10.1098/rspb.2023.0205 PMC9993040 36883277

[21] C. Rochais, T. L. Rymer, N. Pillay, Challenges in linking cognition and survival: A review. Front. Ecol. Evol. 10, 729546 (2022).

[22] R. Croston, C. L. Branch, D. Y. Kozlovsky, R. Dukas, V. V. Pravosudov, Heritability and the evolution of cognitive traits. Behav. Ecol. 26, 1447–1459 (2015).

[23] J. M. S. Burger, M. Kolss, J. Pont, T. J. Kawecki, Learning ability and longevity: A symmetrical evolutionary trade-off in *Drosophila*. Evolution 62, 1294–1304 (2008). 18363867 10.1111/j.1558-5646.2008.00376.x

[24] L. J. Evans, K. E. Smith, N. E. Raine, Fast learning in free-foraging bumble bees is negatively correlated with lifetime resource collection. Sci. Rep. 7, 496 (2017). 28356567 10.1038/s41598-017-00389-0 PMC5428240

[25] M. C. Tello-Ramos, C. L. Branch, D. Y. Kozlovsky, A. M. Pitera, V. V. Pravosudov, Spatial memory and cognitive flexibility trade-offs: To be or not to be flexible, that is the question. Anim. Behav. 147, 129–136 (2019).

[26] C. Soravia, B. J. Ashton, A. Thornton, A. R. Ridley, General cognitive performance declines with female age and is negatively related to fledging success in a wild bird. Proc. R. Soc. B. Biol. Sci. 289, 20221748 (2022). 10.1098/rspb.2022.1748 PMC9768653 36541175

[27] K. B. Sewall, J. A. Soha, S. Peters, S. Nowicki, Potential trade-off between vocal ornamentation and spatial ability in a songbird. Biol. Lett. 9, 20130344 (2013). 23697642 10.1098/rsbl.2013.0344 PMC3730647

[28] N. J. Boogert, J. R. Madden, J. Morand-Ferron, A. Thornton, Measuring and understanding individual differences in cognition. Philos. Trans. R. Soc. Lond. B Biol. Sci. 373, 20170280 (2018). 30104425 10.1098/rstb.2017.0280 PMC6107562

[29] J. M. Burkart, M. N. Schubiger, C. P. van Schaik, The evolution of general intelligence. Behav. Brain Sci. 40, e195 (2017). 27464851 10.1017/S0140525X16000959

[30] R. C. Shaw, M. Schmelz, Cognitive test batteries in animal cognition research: Evaluating the past, present and future of comparative psychometrics. Anim. Cogn. 20, 1003–1018 (2017). 28993917 10.1007/s10071-017-1135-1

[31] M.-A. Poirier, D. Y. Kozlovsky, J. Morand-Ferron, V. Careau, How general is cognitive ability in non-human animals? A meta-analytical and multi-level reanalysis approach. Proc. R. Soc. B Biol. Sci. 287, 20201853 (2020). 10.1098/rspb.2020.1853 PMC7739923 33290683

[32] J. Amici, J. Call, F. Aureli, Coexistence of general intelligence and specialized modules. Behav. Brain Sci. 40, e196 (2017). 29342657 10.1017/S0140525X16001576

[33] J. O. van Horik, E. J. G. Langley, M. A. Whiteside, J. R. Madden, Differential participation in cognitive tests is driven by personality, sex, body condition and experience. Behav. Proc. 134, 22–30 (2017). 10.1016/j.beproc.2016.07.001 27397575

[34] C. Fichtel, K. Dinter, P. M. Kappeler, The lemur baseline: How lemurs compare to monkeys and apes in the Primate Cognition Test Battery. PeerJ 8, e10025-26 (2020). 33024643 10.7717/peerj.10025 PMC7520086

[35] J. Isden, C. Panayi, C. Dingle, J. Madden, Performance in cognitive and problem-solving tasks in male spotted bowerbirds does not correlate with mating success. Anim. Behav. 86, 829–838 (2013).

[36] A. Medina-García, T. F. Wright, An integrative measure of cognitive performance, but not individual task performance, is linked to male reproductive output in budgerigars. Sci. Rep. 11, 11775 (2021). 34083674 10.1038/s41598-021-91213-3 PMC8175410

[37] J. Keagy, J.-F. Savard, G. Borgia, Complex relationship between multiple measures of cognitive ability and male mating success in satin bowerbirds, *Ptilonorhynchus violaceus*. Anim. Behav. 81, 1063–1070 (2011).

[38] S. L. Bressler, V. Menon, Large-scale brain networks in cognition: Emerging methods and principles. Trends Cog. Sci. 14, 277–290 (2010). 10.1016/j.tics.2010.04.004 20493761

[39] R. I. M. Dunbar, S. Shultz, Why are there so many explanations for primate brain evolution? Philos. Trans. R. Soc. Lond. B Biol. Sci. 372, 20160244 (2017). 28673920 10.1098/rstb.2016.0244 PMC5498304

[40] K. R. L. Janmaat, What animals do not do or fail to find: A novel observational approach for studying cognition in the wild. Evol. Anthropol. 28, 303–320 (2019). 31418959 10.1002/evan.21794 PMC6916178

[41] C. Fichtel, Cognition in wild lemurs. Curr. Op. Behav. Sci. 45, 101135 (2022).

[42] R. A. Harrison, E. van de Waal, The unique potential of field research to understand primate social learning and cognition. Curr. Op. Behav. Sci. 45, 101132 (2022).

[43] J. Henke-von der Malsburg, P. Kappeler, C. Fichtel, Linking cognition to ecology in wild sympatric mouse lemur species. Proc. Roy. Soc. Lond. B 288, 20211728 (2021). 10.1098/rspb.2021.1728 PMC8611352 34814746

[44] M. N. Schubiger, Validity of cognitive tests for non-human animals: Pitfalls and prospects. Front. Psychol. 11, 1835 (2020). 32982822 10.3389/fpsyg.2020.01835 PMC7488350

[45] C. Ezran, C. J. Karanewsky, J. L. Pendleton, A. Sholtz, M. R. Krasnow, J. Willick, A. Razafindrakoto, S. Zohdy, M. A. Albertelli, M. A. Krasnow, The mouse lemur, a genetic model organism for primate biology, behavior, and health. Genetics 206, 651–664 (2017). 28592502 10.1534/genetics.116.199448 PMC5499178

[46] C. L. A. Ho, C. Fichtel, D. Huber, The gray mouse lemur (*Microcebus murinus*) as a model for early primate brain evolution. Curr. Op. Neurobiol. 71, 92–99 (2021). 34768148 10.1016/j.conb.2021.09.012

[47] C. Fichtel, Predation in the dark: Antipredator strategies of Cheirogaleidae and other nocturnal primates, in *The Dwarf and Mouse Lemurs of Madagascar. Biology, Behavior and Conservation Biogeography of the Cheirogaleidae*, S. Lehman, U. Radespiel, E. Zimmermann, Eds. (Cambridge Univ. Press, 2016), pp. 366–380.

[48] A. M. Hämäläinen, M. Dammhahn, F. Aujard, M. Eberle, I. Hardy, P. M. Kappeler, M. Perret, S. Schliehe-Diecks, C. Kraus, Senescence or selective disappearance? Age trajectories of body mass in wild and captive populations of a small-bodied primate. Proc. R. Soc. Biol. Sci. Lond. 281, 20140830-10 (2014). 10.1098/rspb.2014.0830 PMC4132673 25100693

[49] J. H. Rakotoniaina, P. M. Kappeler, E. Kaesler, A. M. Hämäläinen, C. Kirschbaum, C. Kraus, Hair cortisol concentrations correlate negatively with survival in a wild primate population. BMC Ecol. 17, 30 (2017). 28859635 10.1186/s12898-017-0140-1 PMC5579956

[50] E. Herrmann, M. V. Hernandez-Lloreda, J. Call, B. Hare, M. Tomasello, The structure of individual differences in the cognitive abilities of children and chimpanzees. Psychol. Sci. 21, 102–110 (2010). 20424030 10.1177/0956797609356511

[51] W. D. Hopkins, J. L. Russell, J. Schaeffer, Chimpanzee intelligence is heritable. Curr. Biol. 24, 1–4 (2014). 25017206 10.1016/j.cub.2014.05.076 PMC4108509

[52] L. A. Damerius, J. M. Burkart, M. A. V. Noordwijk, D. B. M. Haun, Z. K. Kosonen, B. M. F. Galdikas, Y. Saraswati, D. Kurniawan, C. P. van Schaik, General cognitive abilities in orangutans (*Pongo abelii* and *Pongo pygmaeus*). Intelligence 74, 3–11 (2018).

[53] J. O. van Horik, E. J. G. Langley, M. A. Whiteside, P. R. Laker, J. R. Madden, Intra-individual variation in performance on novel variants of similar tasks influences single factor explanations of general cognitive processes. R. Soc. Op. Sci. 5, 171919 (2018). 10.1098/rsos.171919 PMC6083680 30109047

[54] Y. Chaudron, F. Pifferi, F. Aujard, Overview of age-related changes in psychomotor and cognitive functions in a prosimian primate, the gray mouse lemur (*Microcebus murinus*): Recent advances in risk factors and antiaging interventions. Am. J. Primatol. 83, e23337 (2021). 34706117 10.1002/ajp.23337

[55] A. Sih, M. D. Giudice, Linking behavioural syndromes and cognition: A behavioural ecology perspective. Philos. Trans. R. Soc. Lond. B Biol. Sci. 367, 2762–2772 (2012). 22927575 10.1098/rstb.2012.0216 PMC3427552

[56] A. S. Griffin, M. C. Diquelou, Innovative problem solving in birds: A cross-species comparison of two highly successful passerines. Anim. Behav. 100, 84–94 (2015).

[57] J.-M. Gaillard, M. Festa-Bianchet, N. G. Yoccoz, A. Loison, C. Toïgo, Temporal variation in fitness components and population dynamics of large herbivores. Annu. Rev. Ecol. Syst. 31, 367–393 (2000).

[58] L. K. Arvidsson, E. Matthysen, Individual differences in foraging decisions: Information-gathering strategies or flexibility? Behav. Ecol. 27, 1353–1361 (2016).

[59] S. M. Troxell-Smith, V. S. A. Mella, You are what you eat: The interplay between animal personality and foraging ecology. Pers. Nonhuman Anim., 295–305 (2017).

[60] M. Dammhahn, Are personality differences in a small iteroparous mammal maintained by a life-history trade-off? Proc. R. Soc. Lond. B. Biol. Sci. 279, 2645–2651 (2012). 10.1098/rspb.2012.0212 PMC3350711 22398164

[61] C. Kraus, M. Eberle, P. M. Kappeler, The costs of risky male behaviour: Sex differences in seasonal survival in a small sexually monomorphic primate. Proc. R. Soc. Lond. B. Biol. Sci. 275, 1635–1644 (2008). 10.1098/rspb.2008.0200 PMC2602817 18426751

[62] B. R. Smith, D. T. Blumstein, Fitness consequences of personality: A meta-analysis. Behav. Ecol. 19, 448–455 (2007).

[63] L. Z. Garamszegi, M. Eens, J. Torok, Behavioural syndromes and trappability in free-living collared flycatchers, *Ficedula albicollis*. Anim. Behav. 77, 803–812 (2009).

[64] M. C. Diquelou, A. S. Griffin, Does trapping catch sociable, exploratory and innovative mynas preferentially? No, but perhaps less fearful ones. Anim. Behav. 188, 13–24 (2022).

[65] N. J. Boogert, T. W. Fawcett, L. Lefebvre, Mate choice for cognitive traits: A review of the evidence in nonhuman vertebrates. Behav. Ecol. 22, 447–459 (2011).

[66] A. Corral-López, N. I. Bloch, A. Kotrschal, W. van der Bijl, S. D. Buechel, J. E. Mank, N. Kolm, Female brain size affects the assessment of male attractiveness during mate choice. Sci. Adv. 3, e1601990 (2017). 28345039 10.1126/sciadv.1601990 PMC5362185

[67] S. Gao, Y. Jin, F. W. Unverzagt, Y. Cheng, L. Su, C. Wang, F. Ma, A. M. Hake, C. Kettler, C. Chen, J. Liu, J. Bian, P. Li, J. R. Murrell, D. O. Clark, H. C. Hendrie, Cognitive function, body mass index and mortality in a rural elderly Chinese cohort. Arch. Publ. Health 72, 9 (2014). 10.1186/2049-3258-72-9 PMC3974191 24666663

[68] M. Kolk, K. Barclay, Cognitive ability and fertility among Swedish men born 1951–1967: Evidence from military conscription registers. Proc. R. Soc. Lond. B. Biol. Sci. 286, 20190359 (2019). 10.1098/rspb.2019.0359 PMC6532519 31064299

[69] S. A. Heldstab, K. Isler, S. M. Graber, C. Schuppli, C. P. van Schaik, The economics of brain size evolution in vertebrates. Curr. Biol. 32, R697–R708 (2022). 35728555 10.1016/j.cub.2022.04.096

[70] A. Kotrschal, B. Rogell, A. Bundsen, B. Svensson, S. Zajitschek, I. Brännström, S. Immler, A. A. Maklakov, N. Kolm, The benefit of evolving a larger brain: Big-brained guppies perform better in a cognitive task. Anim. Behav. 86, e4–e6 (2013). 24109149 10.1016/j.anbehav.2013.07.011 PMC3791419

[71] P. M. Kappeler, C. Fichtel, A 15-year perspective on the social organization and life history of sifaka in Kirindy Forest, in *Long-Term Field Studies of Primates*, P. M. Kappeler, D. P. Watts, Eds. (Springer, 2012), pp. 101–121.

[72] M. Eberle, P. Kappeler, Mouse lemurs in space and time: A test of the socioecological model. Behav. Ecol. Sociobiol. 51, 131–139 (2002).

[73] A. Ozgul, C. Fichtel, M. Paniw, P. M. Kappeler, Destabilizing effect of climate change on the persistence of a short-lived primate. Proc. Natl. Acad. Sci. U.S.A. 120, e2214244120 (2023). 36972440 10.1073/pnas.2214244120 PMC10083614

[74] L. Peichl, A. Kaiser, F. Rakotondraparany, R. R. Dubielzig, S. M. Goodman, P. M. Kappeler, Diversity of photoreceptor arrangements in nocturnal, cathemeral and diurnal Malagasy lemurs. J. Comp. Neurol. 527, 13–37 (2019). 28054342 10.1002/cne.24167

[75] O. Friard, M. Gamba, BORIS: A free, versatile open-source event-logging software for video/audio coding and live observations. Meth. Ecol. Evol. 7, 1325–1330 (2016).

[76] M. E. Wolak, D. J. Fairbairn, Y. R. Paulsen, Guidelines for estimating repeatability. Meth. Ecol. Evol. 3, 129–137 (2011).

[77] F. John, W. Sanford, Multivariate linear models in R, in *An R Companion to Applied Regression* (SAGE Publications, 2011).

[78] W. Forstmeier, H. Schielzeth, Cryptic multiple hypotheses testing in linear models: Overestimated effect sizes and the winner’s curse. Behav. Ecol. Sociobiol. 65, 47–55 (2011). 21297852 10.1007/s00265-010-1038-5 PMC3015194

[79] T. T. M. L. Thomas, Package “survival”. R Top. Doc., 28–33 (2015).

[80] S. Schliehe-Diecks, M. Eberle, P. M. Kappeler, Walk the line—Dispersal movements of gray mouse lemurs (*Microcebus murinus*). Behav. Ecol. Sociobiol. 66, 1175–1185 (2012). 22822289 10.1007/s00265-012-1371-y PMC3397133

