# Supplementary material for: Cognitive performance is linked to fitness in a wild primate
Source: Sci Adv. 2023 Jul 12;9(28):eadf9365. doi: 10.1126/sciadv.adf9365 (PMC10337904; doi:10.1126/sciadv.adf9365)
Supplement: Supplementary file 1 — Figs. S1 to S3 Tables S1 to S3 Legends for movies S1 to S5 [file sciadv.adf9365_sm.pdf]

Science Advances
AAAS

# Supplementary Materials for

## Cognitive performance is linked to fitness in a wild primate

Claudia Fichtel *et al*.

Corresponding author: Claudia Fichtel, claudia.fichtel@gwdg.de

*Sci. Adv.* **9**, eadf9365 (2023)
DOI: 10.1126/sciadv.adf9365

**The PDF file includes:**

Figs. S1 to S3
Tables S1 to S3
Legends for movies S1 to S5

**Other Supplementary Material for this manuscript includes the following:**

Movies S1 to S5

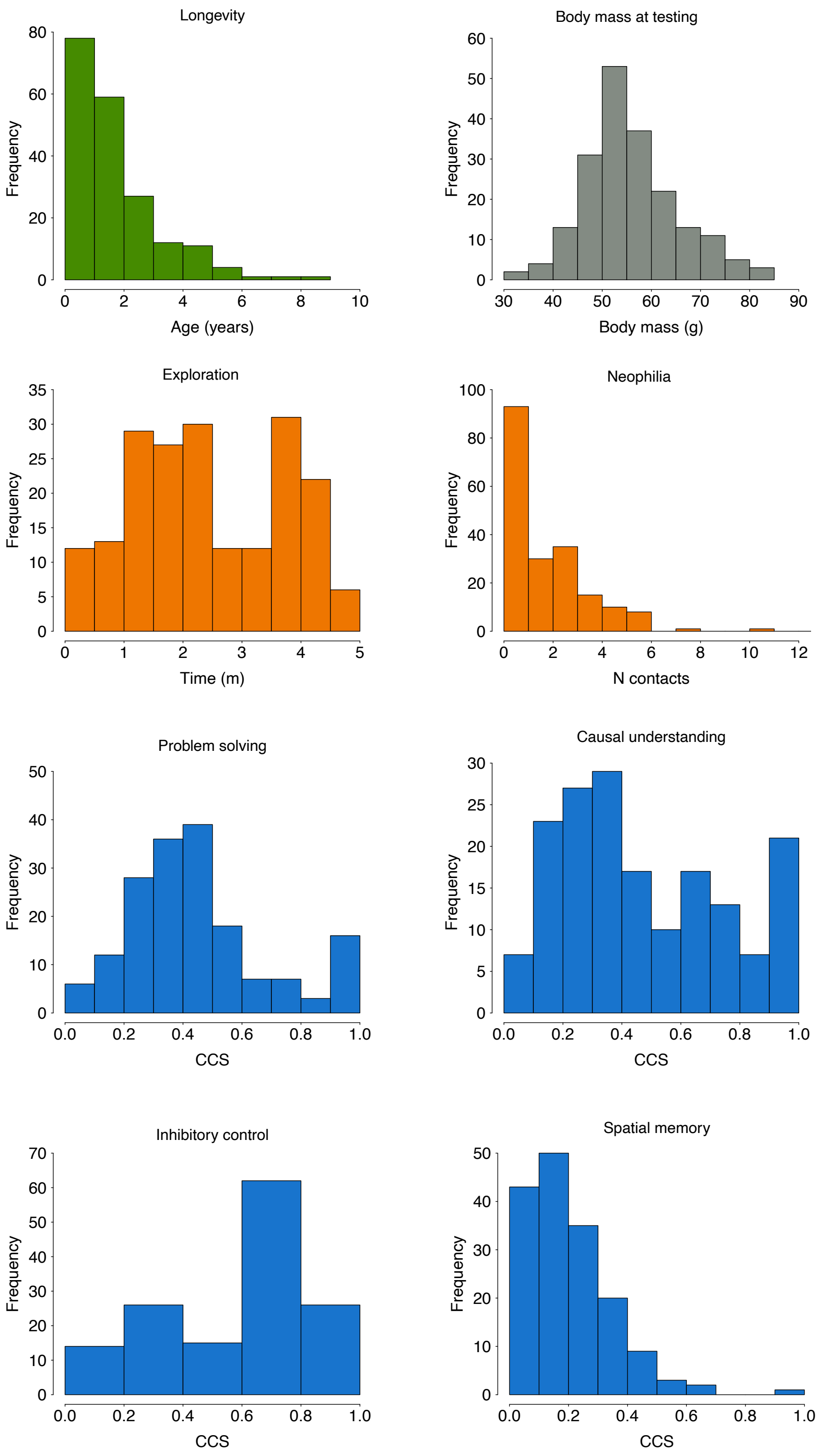


**Fig. S1.** Variation in age, body mass at testing, performance in personality and cognitive tests. Performance in cognitive tests is depicted as cognition score ranging from 0 (good performance) to 1 (poor performance).

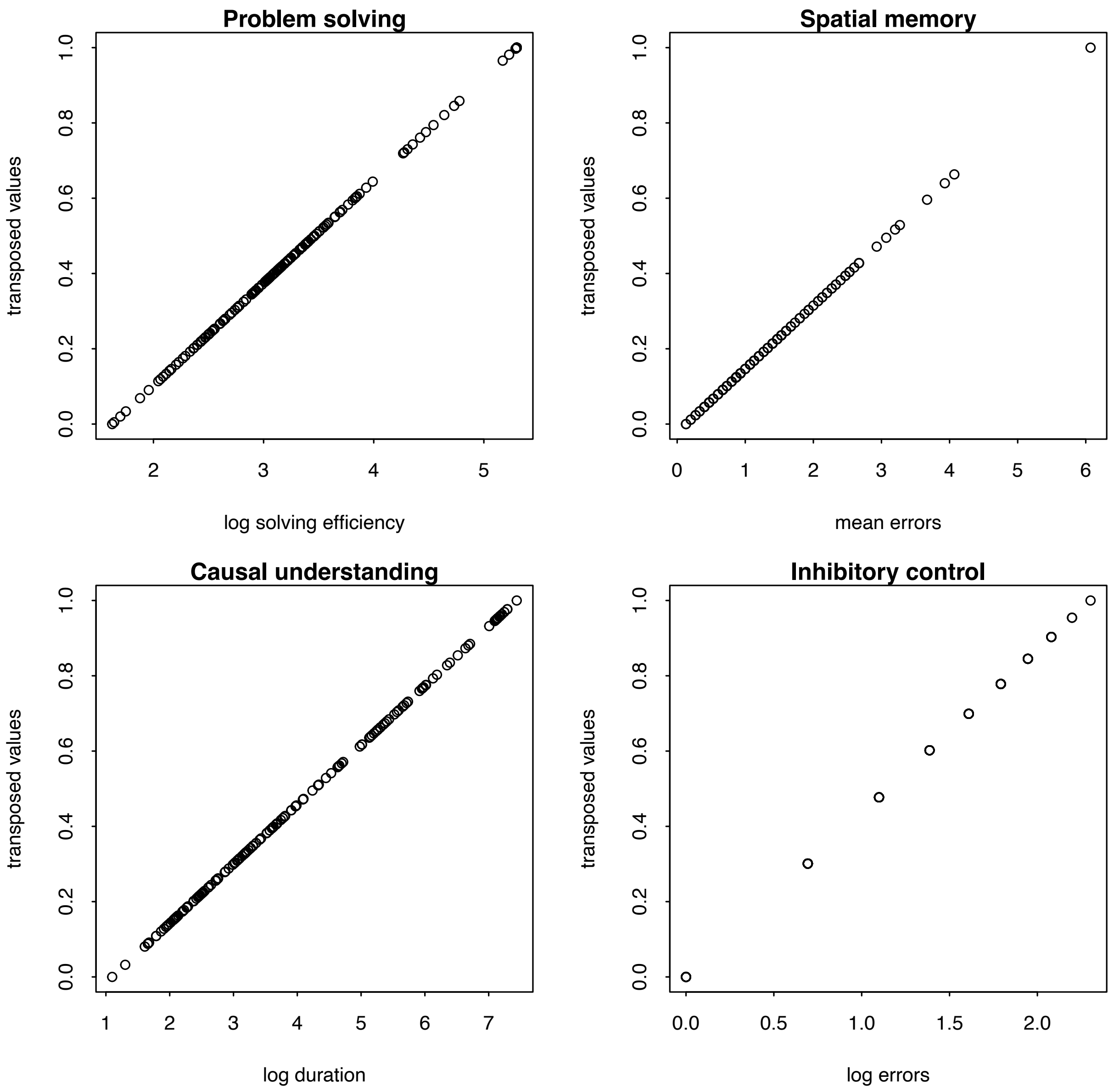


**Fig. S2.** Original values (i.e. frequencies or durations) of performance in the four cognitive tests plotted against the transposed values ranging from 0 (good performance) to 1 (bad performance).

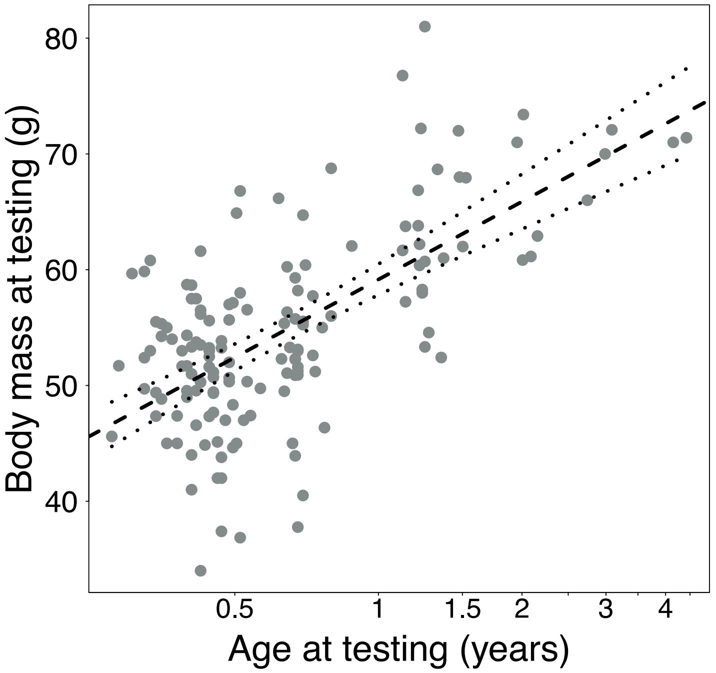


**Fig. S3.** Body mass at testing as a function of age at testing. Dashed lines indicate regression line and its 955% CI.

**Table S1.** Cognitive and personality tests testing cognitive abilities and personality factors that play a pivotal role in fitness-related behaviors

| Test | Associated behaviors |
|---|---|
| Problem solving | Behavioral flexibility |
| Spatial memory | Spatial orientation, navigation |
| Causal understanding | Foraging efficiency |
| Inhibitory control | Self-control beneficial for foraging, predator avoidance, mating |
| Exploration | Information gathering in a new environment |
| Neophilia | Information gathering about new objects |

**Table S2.** Results of the Spearman rank correlation tests across performance in the problem solving, spatial memory, causal understanding, and inhibitory control task.

| **Task** | Problem solving | Spatial memory | Causal understanding |
|---|---|---|---|
| Problem solving | - | - | - |
| Spatial memory | r=0.06, N=142, p=0.454 | - | - |
| Causal understanding | r=0.07, N=144, p=0.426 | r=-0.08, N=143, p=0.322 | - |
| Inhibitory control | r=-0.07, N=134, p=0.388 | r=0.02, N=133, p=0.823 | r=-0.16, N=135, p=0.069 |

**Table S3.** Results of the Cox proportional hazard model estimating the influence sex, CCS, body mass at a testing, exploration, age at testing, neophilia, and average rainfall on survival after testing for individuals which performed all 4 tests (N=130; Wald test:$x^2$=21.01, df=7, p=0.004).

| Term | Coefficient | SE | P |
|---|---|---|---|
| Sex (male)[a] | 0.53 | 0.21 | **0.013** |
| CCS | 0.34 | 0.15 | **0.026** |
| Body mass at testing | -0.35 | 0.15 | **0.015** |
| Exploration | -0.27 | 0.15 | 0.066 |
| Age at testing | -0.11 | 0.15 | 0.481 |
| Neophilia | 0.15 | 0.11 | 0.168 |
| Rainfall | 0.21 | 0.16 | 0.200 |

[a]females as reference category

**Movie S1.**
Mouse lemur performing the problem-solving test.

**Movie S2.**
Mouse lemur performing the spatial memory test.

**Movie S3.**
Mouse lemur performing the causal understanding task.

**Movie S4.**
Mouse lemur performing the inhibitory control task.

**Movie S5.**
Mouse lemur performing the open field and novel object test.
